# Complete genome sequence of *Caballeronia* sp. strain HLA56—a bacterial symbiont isolated from midgut crypts of the leaf-footed bug *Hygia lativentris*

**DOI:** 10.1128/mra.00603-25

**Published:** 2025-08-27

**Authors:** Antoine-Olivier Lirette, Kota Ishigami, Tsubasa Ohbayashi, Yoshitomo Kikuchi

**Affiliations:** 1 Graduate School of Agriculture, Hokkaido University 12810, Sapporo, Japan; 2 Bioproduction Research Institute, National Institute of Advanced Industrial Science and Technology (AIST), Hokkaido Center 74009, Sapporo, Japan; 3 Entomological Laboratory, College of Agriculture, University of the Ryukyus 26428, Nishihara, Okinawa, Japan; 4 Institute for Agro-Environmental Sciences (NIAES), National Agriculture and Food Research Organization (NARO) 13516, Tsukuba, Japan; Indiana University, Bloomington, Bloomington, Indiana, USA

**Keywords:** symbiotic bacteria, symbiosis, insect symbiont

## Abstract

*Caballeronia* sp. strain HLA56 is a bacterial symbiont belonging to the Coreoidea clade in the genus of *Caballeronia*, isolated from the midgut crypts of phytophagous stink bug *Hygia lativentris*. Here, we report the complete 7.78 Mb genome of this symbiont, which consists of six circular replicons containing 7,095 protein-coding genes.

## ANNOUNCEMENT

Various stinkbug species of the infraorder Pentatomomorpha develop specialized symbiotic organs, called crypts, in the posterior midgut, wherein symbiotic bacteria of the genus *Caballeronia* are harbored (1–3). Here, we report the newly sequenced complete genome of a *Caballeronia* symbiont, strain HLA56, isolated from *Hygia lativentris*.

All methods were performed following manufacturer’s instructions unless otherwise stated. HLA56 was isolated from the midgut crypts of an adult *H. lativentris* (Coreoidea: Coreidae) collected on 7 May 2007, in Tsukuba, Ibaraki, Japan (2). The gut symbiotic organ was extracted by forceps under a dissection microscope (Leica S8APO, Germany), washed, and homogenized in sterilized water. The homogenate was spread on YG agar plates (0.5% yeast extract, 0.4% glucose, 0.1% NaCl, and 1.5% agar) and incubated at 26°C for 3 days or longer. The colonies obtained were identified by 16S rRNA sequencing and preserved in 20% glycerol. Genomic DNA was extracted from an overnight culture of glycerol-recovered bacteria in liquid YG medium at 30°C using Genomic-tip 20/G (Qiagen, Germany) and sent to the Bioengineering Lab. Co., Ltd. (Kanagawa, Japan) for sequencing on the Pacific Biosciences Sequel IIe system (PacBio, US) using HiFi sequencing chemistry. The DNA concentration was measured using the QuantiFluor dsDNA System and a Quantus Fluorometer (Promega, US), and the quality assessment was performed by electrophoresis using the 5200 Fragment Analyzer System and Agilent HS Genomic DNA 50 kb Kit (Agilent Technologies, US). The DNA was fragmented to approximately 10–20 kbp using g-TUBE (Covaris, US), and purification was performed by adding 1.5× the volume of AMPure XP (Beckman Coulter, US). Library preparation was performed using SMRTbell Express Template Prep Kit 2.0 (PacBio), and polymerase complexes were formed using the Binding Kit 2.2 (PacBio, US).

Adapter sequences were removed using SMRT Link v11.0.0.146107, and the resulting subreads were aligned to generate circular consensus sequences (CCS). To generate HiFi reads, CCS with an average quality score lower than 20/read were removed. HiFi reads shorter than 1,000 bases were removed using Filtlong v. 0.2.1 (4), representing error-corrected reads. Overall, 22,234 HiFi reads were obtained with an N50 of 1,408,067 bp. High-quality HiFi reads were assembled, and the overlaps were identified and trimmed using Flye v. 2.9.1-b1780 (5) without rotation. Assembled contig graphs were visualized in Bandage v. 0.8.1 (6) to confirm circularity. Genome completeness was confirmed using CheckM2 v. 1.2.2 (7), and annotation was performed using DFAST v1.6.0 (8).

The complete genome of strain HLA56 is 7,788,499 bp (31.7×, GC content: 61.4%, completeness: 99.8%, and contamination: 2.47%) and consists of six circular replicons containing 7,095 protein-coding genes, 10 rRNAs, and 65 tRNAs. The values of pairwise digital DNA-DNA hybridization (dDDH) against other *Caballeronia* symbionts (formerly *Burkholderia*) calculated using ggdc (9) were 35.1% for *Caballeronia insecticola* RPE64 (10) and 29.6% for *Caballeronia cordobensis* RPE67 (11). According to the recommended threshold for dDDH of 70% (12), strain HLA56 is a different species from either *Caballeronia* symbionts isolated from *Riptortus pedestris*. Due to the effects these symbionts have on the lifecycle of their hosts, which are often agricultural pests, their characterization is paramount and has practical pest management applications.

## Data Availability

The genome sequence of Caballeronia sp. HLA56 has been deposited in DDBJ under the accession numbers BAAHOB010000001–BAAHOB010000006. The raw sequences have been deposited in the DDBJ Sequence Read Archive under the accession number DRA021405.
